# Geriatric nutritional risk index predicts cancer prognosis in patients with local advanced rectal cancer undergoing chemoradiotherapy followed by curative surgery

**DOI:** 10.1186/s12957-021-02139-z

**Published:** 2021-01-30

**Authors:** Shozo Ide, Yoshinaga Okugawa, Yusuke Omura, Akira Yamamoto, Takashi Ichikawa, Takahito Kitajima, Tadanobu Shimura, Hiroki Imaoka, Hiroyuki Fujikawa, Hiromi Yasuda, Takeshi Yokoe, Yoshiki Okita, Masaki Ohi, Yuji Toiyama

**Affiliations:** Department of Gastrointestinal and Pediatric Surgery, Mie University Graduate School of Medicine, 2-174 Edobashi, Tsu, Mie 514-8507 Japan

**Keywords:** Rectal cancer, Geriatric nutritional index, Chemoradiotherapy, Prognosis

## Abstract

**Aim:**

The clinical significance of the geriatric nutritional risk index (GNRI) in locally advanced rectal cancer (LARC) patients undergoing preoperative chemoradiotherapy (CRT) followed by curative surgery has not been comprehensively evaluated.

**Methods:**

This retrospective study enrolled 93 LARC patients diagnosed with clinical lymph node metastasis. The GNRI formula was as follows: 1.489 × albumin (g/l) + 41.7 × current weight/ideal weight. Patients were categorized as GNRI low (GNRI < 104.25) or high (GNRI > 104.25) according to the receiver operating characteristic (ROC) curve for survival analysis. The impact of GNRI status on the prognostic outcomes of curative surgery for LARC was examined.

**Results:**

There were 55 (59.14%) and 38 (40.86%) patients in the GNRI high and low groups, respectively. Of the investigated demographic factors, age, pathological tumor invasion, and presence of recurrence were significantly associated with the GNRI value. In Kaplan–Meier analysis, overall survival (OS) and disease-free survival (DFS) were significantly shorter in the GNRI low group (OS: *p* = 0.00020, DFS: *p* = 0.0044, log-rank test). Multivariate analysis using a Cox proportional hazards model showed that a low GNRI was an independent risk factor for poor OS (hazard ratio (HR) = 3.22; 95% confidence interval (CI), 1.37–8.23; *p* = 0.0068) and DFS (HR = 2.32; 95%CI = 1.15–4.79; *p* = 0.018). Although use of adjuvant therapy has no impact on prognosis (OS: *p* = 0.26, DFS: *p* = 0.29), low GNRI showed shorter OS and DFS in patients with pathological lymph node metastasis [ypN(+)] (OS: *p* = 0.033, DFS: *p* = 0.032, log-rank test).

**Conclusions:**

GNRI is a useful marker for LARC patients diagnosed with clinical lymph node metastasis and treated by preoperative CRT followed by curative surgery. GNRI is a useful tool to identify high risk of recurrence for improving the survival in LARC patients.

**Supplementary Information:**

The online version contains supplementary material available at 10.1186/s12957-021-02139-z.

## Introduction

Malnutrition as a risk factor for postoperative complications and worse prognoses in cancer patients has been gradually highlighted [1, 2]. Pretreatment malnutrition also predicts treatment tolerance and toxicity in patients administered chemotherapy and chemoradiotherapy (CRT), and early nutritional intervention provides beneficial outcomes to patients by maintaining their nutritional status and enhancing CRT treatment tolerance [3, 4]. The European Society for Clinical Nutrition and Metabolism (ESPEN) recommends screening all cancer patients for nutritional risk early in the course of their care [5, 6]. The geriatric nutritional risk index (GNRI) is a nutritional screening index of nutrition-related risk associated with the severity of malnutrition and mortality of hospitalized elderly patients [7]. The GNRI is calculated using serum albumin levels and the ratio of current body weight to ideal body weight. The GNRI is associated with prognosis in hemodialysis patients and those with heart failure and cholecystitis [8–12]. In addition, the relationship between the GNRI and prognostic outcomes in patients with malignancies was recently reported [13–18]. Furthermore, low GNRI was useful identifier for high-risk group of morbidity and mortality in elderly patients with colorectal cancer after curative surgery [19, 20].

Preoperative CRT is widely used for local advanced rectal cancer (LARC) to decrease local recurrence and increase the sphincter preservation rate [21, 22]. However, this approach has not improved the rate of distant recurrence, which is now the major cause of death in LARC patients. In addition, the efficacy of adjuvant chemotherapy in patients with rectal cancer receiving preoperative CRT remains controversial [23, 24]. Therefore, the identification of predictive factors for poor prognosis (high risk of recurrence) and the introduction of new advanced treatments are important for LARC patients.

The present study aimed to investigate whether the GNRI is a reliable predictor of the prognostic outcome in LARC patients with suspected clinical lymph node metastasis undergoing CRT followed by rectal cancer resection.

## Methods

### Patients

Ninety-three LARC patients who underwent preoperative CRT followed by rectal cancer resection in Mie University Hospital (Tsu, Japan) between January 2001 and December 2019 were retrospectively analyzed. The criteria for preoperative CRT were as follows: Patients who had clinical stage III based on the International Union Against Cancer TNM classification with an Eastern Cooperative Oncology Group Performance Status of 0 or 1 [25]. In this retrospective study, consecutive patients who were diagnosed clinically with T3 or T4, N1-3, M0 low rectal cancer were enrolled.

### CRT schedules and surgery

LARC patients underwent long-course (a dose of 45 Gy in 25 fractions for 4 weeks) or short-course (a dose of 20 Gy in four fractions for 1 week) radiotherapy using the 4-field approach. All patients received concurrent 5-fluorouracil (5-FU)-based chemotherapy, including 5-FU/leucovorin, tegafur/uracil, capecitabine, and S-1. The time interval between preoperative CRT and surgery was 4–6 weeks for long-course and 2–3 weeks for short-course irradiation. After resection of the tumor, all specimens were analyzed for pathological TNM classification, and staging was determined according to the classification established by the American Joint Committee on Cancer [25]. The degree of histopathological tumor regression was defined based on the Guidelines for Clinical and Pathological Studies on Carcinoma of the Colon and Rectum and classified into 5 grades: grade 0, no necrosis or regressive change; grade 1a, 66% vital residual tumor cells (VRTCs); grade 1b, ~ 33–66% VRTCs; grade 2, < 33% VRTCs; and grade 3, no VRTCs [26]. We defined responders as those with grades 2 and 3 and non-responders as patients with grades 0–1b. 5-FU-based adjuvant chemotherapy was administered following surgery for 6 months to 1 year according to the pathological staging. Follow-up evaluations were performed every 3 months for the first year, every 6 months until the 5 years, and yearly thereafter. Follow-up was performed until patient death, or December 2019, which was the cutoff date for this study.

### Nutritional assessment

The GNRI formula was as follows: GNRI = (1.489 × albumin, g/l) + (41.7 × current/ideal body weight). We computed ideal body weight as the weight corresponding to an ideal body mass index of 22 kg/m^2^. Therefore, the ideal body weight was defined as (height [m])^2^ × 22. As additional nutrition factors, we also measured the platelet-lymphocyte ratio (PLR), prognostic nutrition index (PNI), and modified Glasgow Prognostic Score (mGPS). Height, body weight, and blood samples were obtained from each patient within 1 week prior to nCRT. For the PLR, patients were categorized according to ratios of ≤ 150 or > 150 [27]. For the PNI, patients were divided into two groups using the best cutoff value for survival.

### Statistical analyses

All statistical analyses were performed using JMP version 10 (SAS Institute, Cary, NC, USA). Associations between the GNRI and clinicopathological factors or blood sample tests were analyzed using the Mann–Whitney *U* test. Overall survival (OS) and disease-free survival (DFS) curves were analyzed using the Kaplan–Meier method, and differences were examined using the log-rank test. Univariate and multivariate analyses were performed using the Cox proportional hazards model to determine the factors affecting OS and DFS. Parameters with *p* < 0.05 in the univariate analysis were used for the multivariate analysis. Receiver operating characteristic (ROC) curves were established to determine the cutoff values for prognosis using the Youden index. Probability values less than 0.05 were considered statistically significant.

## Results

### Patient characteristics

**Fig. 1:**
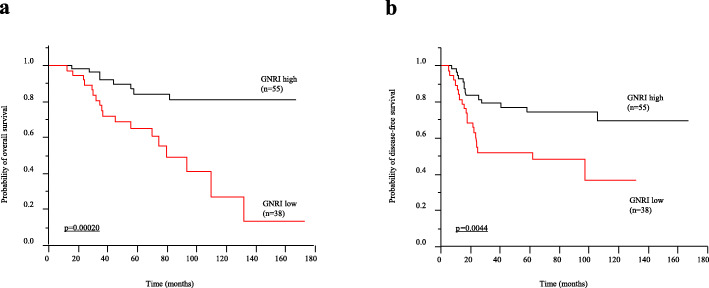
Prognostic impact of the geriatric nutritional risk index (GNRI) prior to chemoradiotherapy (CRT) in patients with rectal cancer. **a** Kaplan–Meier curve for overall survival (OS) in patients with rectal cancer according to pre-CRT GNRI levels (*n* = 93). OS was significantly higher in patients with a high GNRI (*n* = 55) compared with those with a low GNRI (*n* = 38) (*p* = 0.00020, log-rank test). **b** Kaplan–Meier curve for disease-free survival (DFS) in patients with rectal cancer according to pre-CRT GNRI levels (*n* = 93). DFS was significantly higher in patients with a high GNRI (*n* = 55) compared with those with a low GNRI (*n* = 38) (*p* = 0.0044, log-rank test)

Ninety-three patients were enrolled in this study. Serum albumin ranged from 3.1 to 5.0 and average was 4.14 (Supple. Fig. 1a). BMI ranged from 16.53 to 33.05 and average was 23.29 (Supple. Fig. 1b). The GNRI ranged from 79.4 to 128.2 and average was 105.8. The median GNRI was 105.7 and this value had a normal distribution (Supple. Fig. 1c). The median age of study subjects was 63 years (range 32–83 years) and was used as cutoff value of age for analysis. Sixty-eight were males and 25 were females. Their demographic and clinical characteristics are shown in Table 1. The median follow-up period was 60.03 months (range 12–172 months). Eleven patients (12%) experienced local recurrence, and 24 patients (26%) showed distant recurrence. We determined the cutoff values (< 104.25) of the GNRI according to the ROC curve generated for multiple logistic regression analysis using the 5-year OS as the endpoint. Eighteen death events were recorded in the low GNRI group and 8 were recorded in the high group. Twenty recurrence events were recorded in the low GNRI group and 14 were recorded in the high group. The associations between the GNRI and clinicopathological factors are shown in Table 2. The GNRI was significantly associated with age, pathological tumor invasion, and presence of recurrence. In constant, the GNRI exhibited no association with tumor progression (pathological N stage, lymphatic and venous invasion status, and tumor histopathological features), pathological response, radiation type, adjuvant chemotherapy, and tumor markers.

**Table 1:** Characteristics of patients with locally advanced rectal cancer who underwent neoadjuvant chemoradiotherapy

Category		*n* (%)
Age (years)	≤ 63	48 (52%)
	≥ 63	45 (48%)
Sex	Male	68 (73%)
	Female	25 (27%)
Adjuvant therapy	Yes	65 (70%)
	No	28 (30%)
Clinical T stage	T3	68 (73%)
	T4	25 (27%)
Clinical N stage	N1	51 (55%)
	N2	26 (28%)
	N3	16 (17%)
ypT stage	T0/T1	11 (12%)
	T2	28 (30%)
	T3	49 (53%)
	T4	5 (5%)
ypN stage	N0	53 (57%)
	N1–3	40 (43%)
Pathological TNM stage	0/I	29 (31%)
	II	23 (25%)
	III	41 (44%)
Radiotherapy	Short-course (20 Gy/4 fractions)	24 (26%)
	Long-course (45 Gy/25 fractions)	69 (74%)
Pathological response	Non-responder (grade 0/1a/1b)	59 (63%)
	Responder (grade 2/3)	34 (37%)
Histology	Well/moderate	81 (87%)
	Poorly/mucinous/signet	12 (13%)
Recurrence	Absent	58 (62%)
	Local	11 (12%)
	Distant	24 (26%)

*yp* pathological status after neoadjuvant therapy, *TNM* tumor node metastasis

**Table 2:** Association between GNRI and clinicopathological factors in patients with locally advanced rectal cancer undergoing neoadjuvant chemoradiotherapy

Category	GNRI high (> 104.25)	GNRI low (< 104.25)	*P* value
Age, years			
≤63	36	12	0.0013
> 63	19	26	
Sex			
Male	40	28	0.92
Female	15	10	
Pathological T			
pT0-2	28	11	0.035
pT3-4	27	27	
Pathological N			
pN0	31	22	0.88
pN1-2	24	16	
Histology			
Well/moderate	49	32	0.49
Poorly/signet/mucinous	6	6	
Lymphatic invasion			
Absent	31	22	0.88
Present	24	16	
Venous invasion			
Absent	34	23	0.90
Present	21	15	
Pathological response			
Non-responder (grade0/1a/1b)	32	27	0.21
Responder (grade2/3)	23	11	
Radiation			
Short course	14	10	0.93
Long course	41	28	
Adjuvant chemotherapy			
Yes	42	23	0.10
No	13	15	
CA19-9 (ng/ml)			
≤ 37.0	47	28	0.16
> 37.0	8	10	
CEA (ng/ml)			
≤ 5	29	15	0.21
> 5	26	23	
Recurrence			
Absent	40	18	0.040
Local	4	7	
Distant	11	13	

Note: Data in the table are the number of patients in each category

*GNRI* geriatric nutritional risk index, *CA19-9* carbohydrate antigen 19-9, *CEA* carcinoembryonic antigen

### GNRI and oncologic outcome

**Fig. 2:**
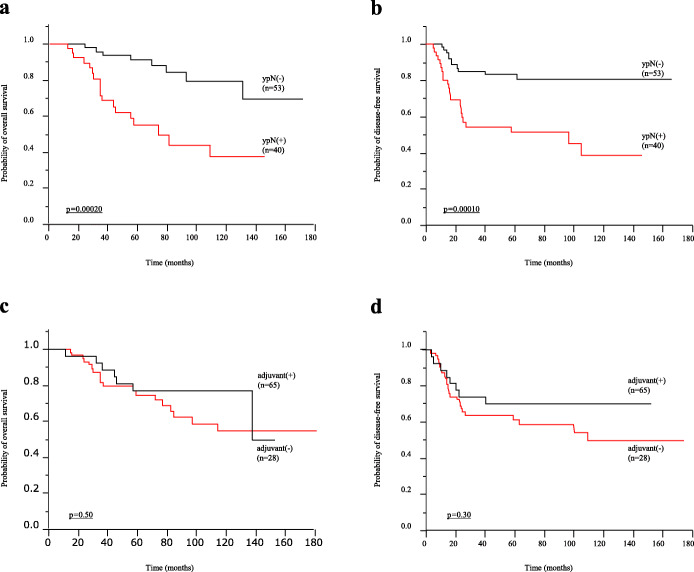
Prognostic impact of pathological lymph node metastasis and adjuvant chemotherapy in patients with rectal cancer. **a** Kaplan–Meier curve for overall survival (OS) in patients with rectal cancer according to the status of pathological lymph node metastasis (*n* = 93). OS was significantly higher in patients with lymph node negative status [ypN(−)] (*n* = 53) compared with those with lymph node positive status [ypN(+)] (*n* = 40) (*p* = 0.00020, log-rank test). **b** Kaplan–Meier curve for disease-free survival (DFS) in patients with rectal cancer according to the status of pathological lymph node metastasis (*n* = 93). DFS was significantly higher in patients with lymph node negative status [ypN(−)] (*n* = 53) compared with those with lymph node positive status [ypN(+)] (*n* = 40) (*p* = 0.00010, log-rank test). **c** Kaplan–Meier curve for OS in patients with rectal cancer according to adjuvant chemotherapy (*n* = 93). OS was not significantly different between adjuvant (+) (*n* = 65) and adjuvant (−) (*n* = 28) (*p* = 0.50, log-rank test). **d** Kaplan–Meier curve for DFS in patients with rectal cancer according to adjuvant chemotherapy (*n* = 93). DFS was not significantly different between adjuvant (+) (*n* = 65) and adjuvant (−) (*n* = 28) (*p* = 0.30, log-rank test)

The Kaplan–Meier analysis showed significantly poorer OS in the GNRI low group than in the high group (*p* = 0.0002) (Fig. 1a). Univariate analysis for OS showed that pathological lymph node metastasis positive (*p* = 0.00040), lymphatic invasion positive (*p* = 0.017), carbohydrate antigen 19-9 (CA19-9) high (*p* = 0.0041), cancer embryonic antigen (CEA) high (*p* = 0.022), and GNRI low (*p* = 0.0003) were risk factors for poor OS. Furthermore, multivariate analysis using a Cox proportional hazards model showed that pathological lymph node metastasis positive [hazard ratio (HR) = 4.15; 95% confidence interval (CI), 1.68–11.05; *p* = 0.0018] and GNRI low (HR = 4.37; 95%CI = 1.88–11.10; *p* = 0.00050) was an independent risk factor for poor OS (Table 3). Likewise, the Kaplan–Meier analysis showed significantly poorer DFS in the GNRI low group than in the high group (*p* = 0.0044) (Fig. 1b). Univariate analysis for DFS showed that pathological lymph node positive (*p* = 0.00020), vascular invasion positive (*p* = 0.0028), radiation effect grades 2 and 3 (*p* = 0.031), CEA high (*p* = 0.044), and GNRI low (*p* = 0.0054) were risk factors for poor DFS. Furthermore, multivariate analysis using a Cox proportional hazards model showed that pathological lymph node positive (HR = 3.28; 95%CI = 1.57–7.27; *p* = 0.0014) and GNRI low (HR = 2.71; 95%CI = 1.36–5.60; *p* = 0.0047) was an independent risk factor for poor DFS (Table 4). On the other hand, there was no difference between the long and short course CRT in terms of GNRI association with prognosis (Supple. Fig. 2a-f).

**Table 3:** Univariate and multivariate analyses of predictive factors associated with overall survival in patients with locally advanced rectal cancer undergoing neoadjuvant chemoradiotherapy

		Univariate analysis			Multivariate analysis		
Variable		HR	95% CI	*p* value	HR	95% CI	*p* value
Sex	Male vs. female	2.67	0.92–11.26	0.072			
Age (years)	> 63 vs. ≤63	1.06	0.49–2.31	0.88			
Histological type	Poor/mucinous vs. well/moderate	1.36	0.40–3.57	0.58			
Pathological N stage	ypN(+) vs ypN(−)	4.23	1.89–10.34	0.00040	4.15	1.68–11.05	0.0018
Lymphatic invasion	2, 3 vs. 0, 1	2.14	0.97–5.03	0.059			
Vascular invasion	2, 3 vs. 0, 1	2.60	1.19–5.83	0.017	1.47	0.61–3.66	0.40
Radiation effect	Grades 2, 3 vs. 0–1	1.91	0.84–4.92	0.13			
Radiation course	Short vs. long	1.72	0.76–3.78	0.19			
Adjuvant chemotherapy	Yes vs. no	1.35	0.59–3.47	0.49			
CA19-9	> 37.0 vs. ≤ 37.0 ng/ml	2.56	1.04–5.75	0.0041	2.00	0.76–4.91	0.15
CEA	> 5 vs. ≤ 5 ng/ml	2.69	1.14–7.36	0.022	1.81	0.73–5.17	0.21
PLR	> 150 vs. ≤ 150	1.07	0.47–2.32	0.87			
PNI	≤ 51 vs. > 51	2.03	0.91–4.96	0.084			
mGPS	1, 2 vs. 0	1.27	0.49–2.92	0.60			
GNRI	≤ 104.25 vs. > 104.25	4.36	1.93–10.77	0.0003	4.37	1.88–11.10	0.00050

Note: Parameters with *p* < 0.05 in the univariate analysis were used for the multivariate analysis

*HR* hazard ratio, *CI* confidence interval, *CA19-9* carbohydrate antigen 19-9, *CEA* carcinoembryonic antigen, *PLR* platelet-lymphocyte ratio, *PNI* prognostic nutrition index, *mGPS* modified Glasgow Prognostic score, *GNRI* geriatric nutritional risk index

**Table 4:** Univariate and multivariate analyses of predictive factors associated with disease-free survival in patients with locally advanced rectal cancer undergoing neoadjuvant chemoradiotherapy

		Univariate analysis			Multivariate analysis		
Variable		HR	95% CI	*p* value	HR	95% CI	*p* value
Sex	Male vs. female	2.30	0.97–6.76	0.060			
Age (years)	> 63 vs. ≤ 63	1.13	0.57–2.22	0.74			
Histological type	Poor/mucinous vs. well/moderate	1.53	0.55–6.37	0.46			
Pathological N stage	ypN(+) vs ypN(−)	3.78	1.88–8.07	0.00020	3.28	1.57–7.27	0.0014
Lymphatic invasion	2, 3 vs. 0, 1	1.54	0.78–3.07	0.21			
Vascular invasion	2, 3 vs. 0, 1	2.84	1.43–5.79	0.0028	1.75	0.84–3.72	0.14
Radiation effect	Grades 2, 3 vs. 0–1	2.28	1.07–5.39	0.031	1.59	0.72–3.90	0.26
Radiation course	Short vs. long	1.25	0.58–2.52	0.55			
Adjuvant chemotherapy	Yes vs. no	1.51	0.72–3.58	0.29			
CA19-9	> 37.0 vs. ≤ 37.0 ng/ml	1.85	0.82–3.84	0.13			
CEA	> 5 vs. ≤ 5 ng/ml	2.05	1.02–4.37	0.044	1.85	0.89–4.06	0.10
PLR	> 150 vs. ≤ 150	1.16	0.57–2.28	0.67			
PNI	≤ 51 vs. > 51	1.56	0.79–3.21	0.20			
mGPS	1, 2 vs. 0	1.03	0.41–2.25	0.94			
GNRI	≤ 104.25 vs. > 104.25	2.65	1.33–5.42	0.0054	2.71	1.36–5.60	0.0047

Note: Parameters with *p* < 0.05 in the univariate analysis were used for the multivariate analysis

*HR* hazard ratio, *CI* confidence interval, *CA19-9* carbohydrate antigen 19-9, *CEA* carcinoembryonic antigen, *PLR* platelet-lymphocyte ratio, *PNI* prognostic nutrition index, *mGPS* modified Glasgow Prognostic score, *GNRI* geriatric nutritional risk index

### A low GNRI predicts poor prognosis and recurrence in patients with pathological lymph node metastasis

**Fig. 3:**
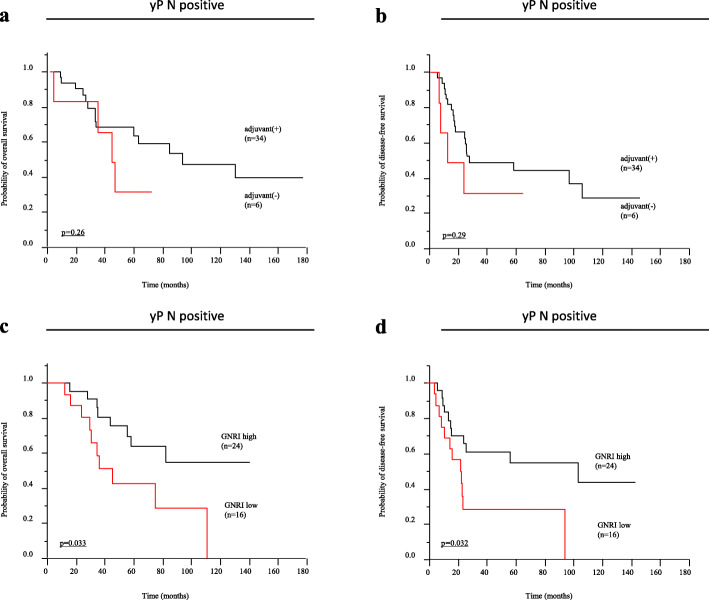
Prognostic impact of adjuvant chemotherapy and the geriatric nutritional risk index (GNRI) in rectal cancer patients with pathological lymph node metastasis. **a** Kaplan–Meier curve for overall survival (OS) in patients with pathological lymph node metastasis according to adjuvant chemotherapy (*n* = 40). OS was not significantly different between adjuvant (+) (*n* = 34) and adjuvant (−) (*n* = 6) (*p* = 0.26, log-rank test). **b** Kaplan–Meier curve for disease-free survival (DFS) in rectal cancer patients with pathological lymph node metastasis according to adjuvant chemotherapy (*n* = 40). DFS was not significantly different between adjuvant (+) (*n* = 34) and adjuvant (−) (*n* = 6) (*p* = 0.29, log-rank test). **c** Kaplan–Meier curve for OS in patients with pathological lymph node metastasis according to GNRI levels (*n* = 40). OS was significantly higher in patients with a high GNRI (*n* = 24) compared with those with a low GNRI (*n* = 16) (*p* = 0.033, log-rank test). **d** Kaplan–Meier curve for DFS in patients with pathological lymph node metastasis according to GNRI levels (*n* = 40). DFS was significantly higher in patients with a high GNRI (*n* = 24) compared with those with a low GNRI (*n* = 16) (*p* = 0.032, log-rank test)

We demonstrated that pathological lymph node metastasis [ypN(+)] predicted poor prognosis and early recurrence in LARC patients (OS: *p* = 0.00020, DFS: *p* = 0.00010) (Fig. 2a and b). In contrast, adjuvant chemotherapy had no impact on prognosis in these patients (OS: *p* = 0.50, DFS: *p* = 0.30) (Figs. 2c and 3d). Next, we analyzed OS and DFS in patients with pathological lymph node metastasis [ypN(+)], and the results showed that adjuvant chemotherapy was not associated with a better prognosis in these patients (OS: *p* = 0.26, DFS: *p* = 0.29) (Fig. 3a and b). In contrast, the GNRI clearly divided the patients into better and poorer prognostic groups (OS: *p* = 0.033, DFS: *p* = 0.032) (Fig. 3c and d).

### Analysis of oncologic outcome using serum albumin or BMI

We analyzed the prognostic significance of serum albumin and BMI, separately. Kaplan–Meier analysis showed that OS and DFS were not significantly different between high and low serum albumin levels (OS: *p* = 0.29, DFS: *p* = 0.25) (Supple. Fig. 3a and 3b). On the other hand, Kaplan–Meier analysis showed that OS was significantly higher in patients with high BMI group compared with those with low group (OS: *p* = 0.021) (Supple. Fig. 3c). However, DFS was not significantly different between high and low BMI group (DFS: *p* = 0.10) (Supple. Fig. 3d).

## Discussion

To the best of our knowledge, this is the first study to investigate the GNRI and clinicopathological factors in LARC patients with suspected clinical lymph node metastasis undergoing CRT followed by curative resection. The current study revealed two significant findings: (1) A low GNRI is an independent predictor of both shorter OS and DFS in LARC patients with clinical lymph node metastasis. (2) A low GNRI is also associated with a significantly worse prognosis and earlier recurrence in patients with pathological lymph node metastasis [ypN(+)].

The GNRI is calculated using serum albumin and current/ideal body weight. Serum albumin and BMI are both definitive factors that can reflect the risk of poor survival and early recurrence in patients with malignancies [28–36]. Although the nutritional status was assessed using various markers, serum albumin is one of the most sensitive and accurate markers for nutritional status. The immune response is directly affected by the nutrition status; thus, a decline in serum albumin leads to immunodeficiency of cell-mediated immunity for the host defenses against cancer [37]. In addition, BMI is related to malnutrition. A low BMI is associated with poor cancer survival because body weight loss is often observed in cases of aggressive cancer or the presence of negative cell regulatory systems for cancer [38]. Moreover, a previous study reported that overweight patients showed biochemical evidence for better nutrition than normal-weight patients because they have more adipose tissue, suggesting that they are less likely to suffer from energy deficits and may have a better tolerance for further postoperative treatment [39, 40]. As a result, body weight loss or a low BMI is considered as a negative prognostic factor for cancer patients. Thus, the combination of serum albumin and BMI increase the power of the GNRI as a prognostic indicator in LARC patients undergoing preoperative CRT followed by curative surgery. In fact, our present study demonstrated that the GNRI is a more effective prognostic marker compared with other nutritional markers by multivariate analysis using a Cox proportional hazards model for OS and DFS. Although past studies demonstrated that GNRI was an independent prognostic factor in patients with colorectal cancer after curative surgery [19, 20], clinical significance of GNRI in patients with LARC undergoing CRT followed by curative surgery has not been mentioned.

The selective use of pelvic CRT following the total mesorectal excision for LARC has dramatically reduced the local recurrence rate from ~ 25% to ~ 5–10% [22, 41, 42]. However, this treatment strategy has not significantly reduced the rate of distant recurrence [42, 43], which is now the major cause of rectal cancer-related death. Risk-adapted alternate strategies are being explored to reduce this recurrence and improve the survival of LARC patients. The use of postoperative adjuvant chemotherapy based on 5-FU or oxaliplatin has not supported the evidence of improved OS or DFS [44]. Recently, total neoadjuvant treatment (TNT), which is intensified neoadjuvant therapy and involves shifting adjuvant chemotherapy to the neoadjuvant setting, was suggested to be more effective for LARC patients with high-risk factors [45–48]. TNT consists of induction chemotherapy, concurrent CRT, and consolidation chemotherapy. A conventional strategy is needed to achieve the appropriate interval between the completion of concurrent CRT and curative surgery. TNT has the advantages of starting systemic chemotherapy 3–4 months earlier than conventional concurrent CRT, which may potentially increase the long-term survival because of the sufficient control of systemic micro-metastasis and improved tolerance to chemo-related toxicities. The present study showed that pathological lymph node metastasis [pN(+)] determined prognosis, but postoperative adjuvant chemotherapy did not contribute to improved prognoses. In contrast, the GNRI was identified as a useful marker to predict survival and recurrence in LARC patients with lymph node metastasis [pN(+)] undergoing CRT followed by curative surgery, which allows for the selection of patients for TNT.

Several limitations of this study should be noted. First, we performed to set up a new cut-off value of GNRI calculated from a ROC curve on the basis of 5-year outcome. Our study differed from past literatures because rectal cancer patients with good performance status who can undergo preoperative chemoradiotherapy followed by surgery were enrolled. As the results, patients in our cohort have high albumin levels and high BMI compared past literatures. Therefore, we considered that it is more rational to set up a new cutoff value of GNRI. Second, our study consisted of a retrospective study design with a relatively small and long-term cohort. For this reason, our study presents several confounding factors related to OS and DFS to reconsider. Third, patient characteristics, such as neoadjuvant CRT regimens, were heterogeneous, and the time intervals between CRT and surgery were inconsistent. Therefore, we need to plan the prospective studies using large cohorts with a longer follow-up and standard pretreatment characteristics to validate these results. We also need to conduct the prospective study to evaluate the prognostic benefit of TNT in low GNRI score.

Fourth, our study included a small number of young patients. GNRI was at first targeted benign diseases (for example, heat failure, hemodialysis, and cholecystitis). Therefore, it was used exclusively by the elderly patients. Our study also demonstrated that GNRI was significantly lower in elderly patients (> 63 years old). However, GNRI is easily calculated using serum albumin, height, and body weight, which are generally measured on admission not only elderly but also younger patients. For this reason, we believed that it was not necessary to limit the target to the elderly patients and that it could be used as a universal marker including younger patients. In fact, there are some reports to evaluate GNRI as prognostic biomarker that included younger patients as well as our study [14, 17, 49].

In conclusion, we identified the GNRI as a significantly independent biomarker of poor prognosis and early recurrence in LARC patients undergoing CRT followed by curative surgery. The GNRI is a convenient decision marker for treatment in LARC patients because it is easy to measure and does not require special techniques or expertise.

## Supplementary Information

**Additional file 1: Supplementary Fig. 1.** (a) The distribution of serum albumin level in our study group. (b) The distribution of body mass index (BMI) in our study group. (c) The distribution of geriatric nutritional risk index (GNRI) in our study group. The GNRI exhibited a normal distribution.

**Additional file 2: Supplementary Fig. 2.** Prognostic impact of radiation course and the geriatric nutritional risk index (GNRI). (a) Kaplan–Meier curve for overall survival (OS) in patients with rectal cancer according to the select of radiation course (*n* = 93). OS was not significantly different between long course chemoradiation (CRT) (*n* = 69) and short course CRT (*n* = 24) (*p* = 0.18, log-rank test). (b) Kaplan–Meier curve for disease-free survival (DFS) in patients with rectal cancer according to the select of radiation course (*n* = 93). DFS was not significantly different between long course chemoradiation (*n* = 69) and short course CRT (*n* = 24) (*p* = 0.24, log-rank test). (c) Kaplan–Meier curve for OS in rectal cancer patients with GNRI high group (*n* = 55). OS was not significantly different between long course CRT (*n* = 41) and short course CRT (*n* = 14) (*p* = 0.23, log-rank test). (d) Kaplan–Meier curve for DFS in rectal cancer patients with GNRI high group (*n* = 55). DFS was not significantly different between long course CRT (*n* = 41) and short course CRT (*n* = 14) (*p* = 0.52, log-rank test). (e) Kaplan–Meier curve for OS in rectal cancer patients with GNRI low group (*n* = 38). OS was not significantly different between long course CRT (*n* = 28) and short course CRT (*n* = 10) (*p* = 0.49, log-rank test). (f) Kaplan–Meier curve for DFS in rectal cancer patients with GNRI low group (*n* = 38). DFS was not significantly different between long course CRT (*n* = 28) and short course CRT (*n* = 10) (*p* = 0.96, log-rank test).

**Additional file 3: Supplementary Fig. 3.** Prognostic impact of serum albumin and body mass index (BMI) in patients with rectal cancer. (a) Kaplan–Meier curve for overall survival (OS) in patients with rectal cancer according to the albumin level (*n* = 93). OS was not significantly different between high level albumin group (*n* = 50) and low level group (*n* = 43) (*p* = 0.29, log-rank test). (b) Kaplan–Meier curve for disease-free survival (DFS) in patients with rectal cancer according to the albumin level (*n* = 93). DFS was not significantly different between high level albumin group (*n* = 50) and low level group (*n* = 43) (*p* = 0.25, log-rank test). (c) Kaplan–Meier curve for overall survival (OS) in patients with rectal cancer according to the BMI (*n* = 93). OS was significantly higher in patients with a high BMI (*n* = 41) compared with those with a low BMI (*n* = 52) (*p* = 0.021, log-rank test). (d) Kaplan–Meier curve for disease-free survival (DFS) in patients with rectal cancer according to the BMI (*n* = 93). DFS was not significantly different between high BMI (*n* = 50) and low BMI (*n* = 43) (*p* = 0.10, log-rank test).

## Data Availability

Primary research data are presented in a summative fashion. No publicly available datasets have been generated as part of this work.

## Abbreviations

CRT: Chemoradiotherapy
ESPEN: European Society for Clinical Nutrition and Metabolism
GNRI: Geriatric nutritional risk index
LARC: Local advanced rectal cancer
5-FU: 5-Fluorouracil
VRTCs: Vital residual tumor cells
PLR: Platelet-lymphocyte ratio
NLR: Neutrophil-lymphocyte ratio
LMR: Lymphocyte-monocyte ratio
AGR: Albumin-globulin ratio
CRP: C-reactive protein
CAR: CRP albumin ratio
PNI: Prognostic nutrition index
mGPS: Modified Glasgow Prognostic Score
OS: Overall survival
DFS: Disease-free survival
ROC: Receiver operating characteristic
CA19-9: Carbohydrate antigen 19-9
CEA: Carcinoma embryonic antigen
HR: Hazard ratio
CI: Confidence interval
BMI: Body mass index
TNT: Total neoadjuvant treatment
