# Supplementary material for: Geriatric nutritional risk index predicts cancer prognosis in patients with local advanced rectal cancer undergoing chemoradiotherapy followed by curative surgery
Source: World J Surg Oncol. 2021 Jan 30;19:34. doi: 10.1186/s12957-021-02139-z (PMC7847581; doi:10.1186/s12957-021-02139-z)
Supplement: Supplementary file 1 — Additional file 1: Supplementary Fig. 1. (a) The distribution of serum albumin level in our study group. (b) The distribution of body mass index (BMI) in our study group. (c) The distribution of geriatric nutritional risk index (GNRI) in our study group. The GNRI exhibited a normal distribution. [file 12957_2021_2139_MOESM1_ESM.pptx]

## Slide 1
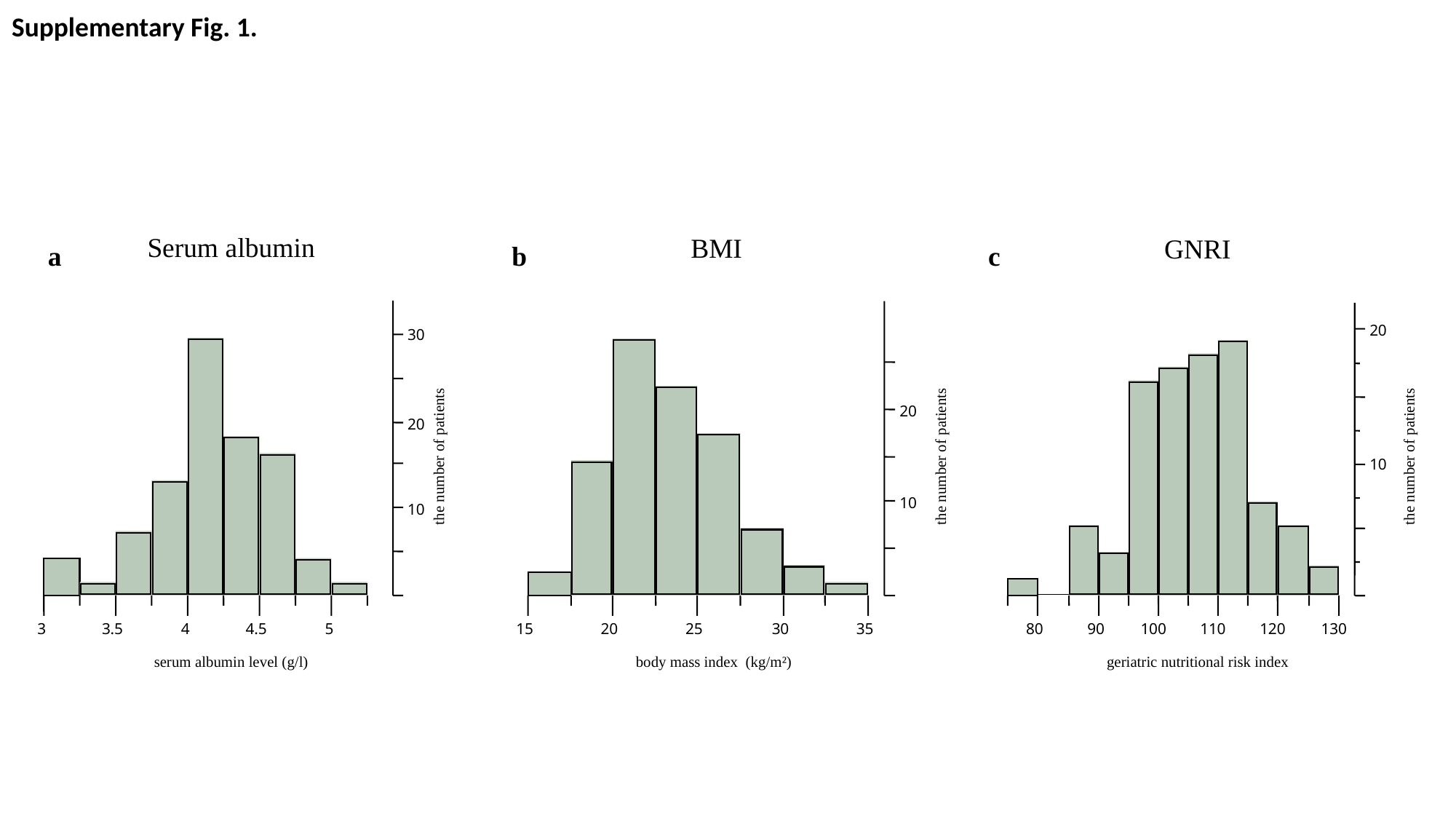

Supplementary Fig. 1.
Serum albumin
BMI
GNRI
a
b
c
30
20
10
3
3.5
4
4.5
5
20
10
15
20
25
30
35
20
10
80
90
100
110
120
130
the number of patients
the number of patients
the number of patients
serum albumin level (g/l)
body mass index (kg/m²)
geriatric nutritional risk index
