# Supplementary material for: Geriatric nutritional risk index predicts cancer prognosis in patients with local advanced rectal cancer undergoing chemoradiotherapy followed by curative surgery
Source: World J Surg Oncol. 2021 Jan 30;19:34. doi: 10.1186/s12957-021-02139-z (PMC7847581; doi:10.1186/s12957-021-02139-z)
Supplement: Supplementary file 2 — Additional file 2: Supplementary Fig. 2. Prognostic impact of radiation course and the geriatric nutritional risk index (GNRI). (a) Kaplan–Meier curve for overall survival (OS) in patients with rectal cancer according to the select of radiation course (n = 93). OS was not significantly different between long course chemoradiation (CRT) (n = 69) and short course CRT (n = 24) (p = 0.18, log-rank test). (b) Kaplan–Meier curve for disease-free survival (DFS) in patients with rectal cancer according to the select of radiation course (n = 93). DFS was not significantly different between long course chemoradiation (n = 69) and short course CRT (n = 24) (p = 0.24, log-rank test). (c) Kaplan–Meier curve for OS in rectal cancer patients with GNRI high group (n = 55). OS was not significantly different between long course CRT (n = 41) and short course CRT (n = 14) (p = 0.23, log-rank test). (d) Kaplan–Meier curve for DFS in rectal cancer patients with GNRI high group (n = 55). DFS was not significantly different between long course CRT (n = 41) and short course CRT (n = 14) (p = 0.52, log-rank test). (e) Kaplan–Meier curve for OS in rectal cancer patients with GNRI low group (n = 38). OS was not significantly different between long course CRT (n = 28) and short course CRT (n = 10) (p = 0.49, log-rank test). (f) Kaplan–Meier curve for DFS in rectal cancer patients with GNRI low group (n = 38). DFS was not significantly different between long course CRT (n = 28) and short course CRT (n = 10) (p = 0.96, log-rank test). [file 12957_2021_2139_MOESM2_ESM.pptx]

## Slide 1
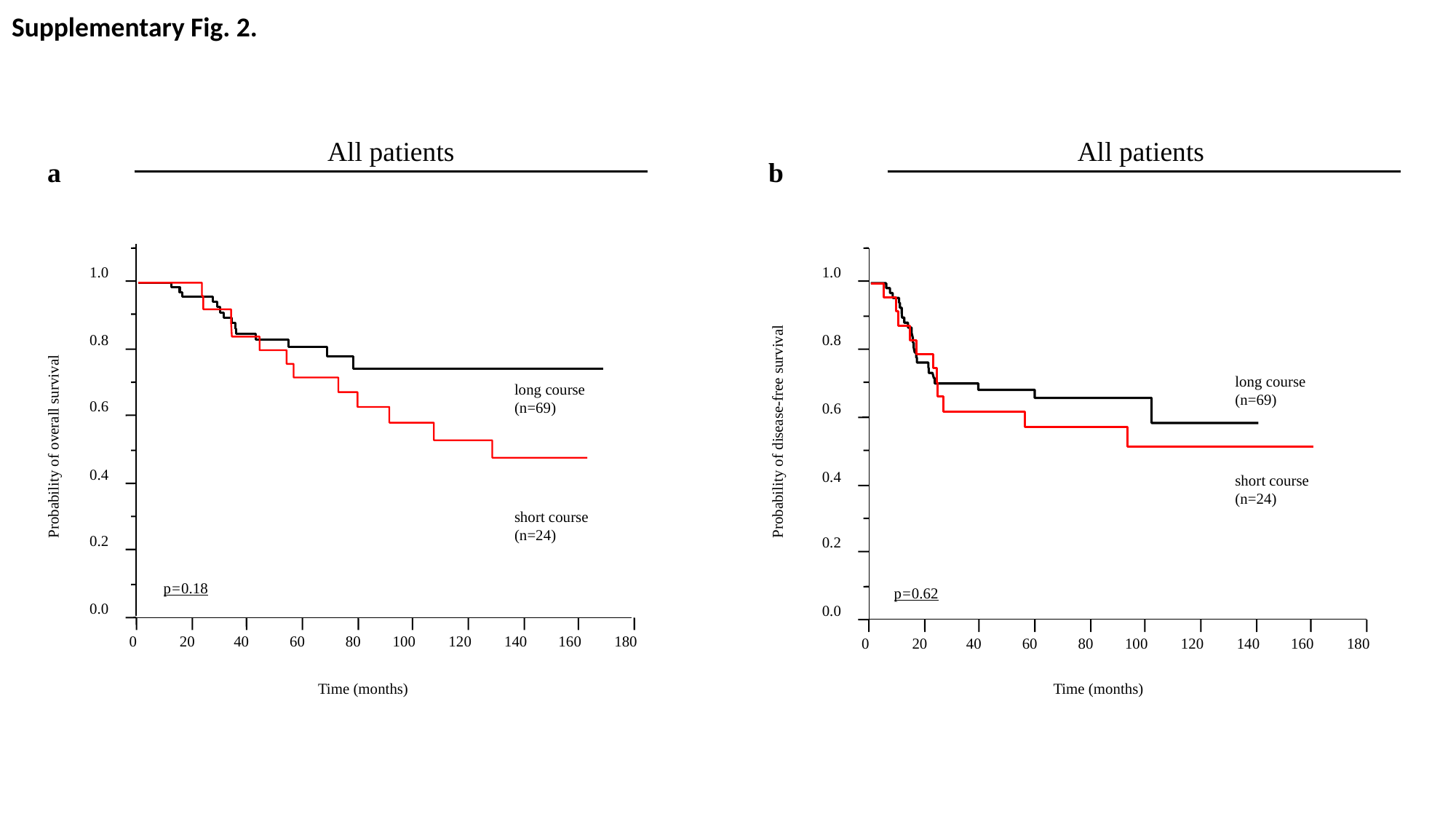

Supplementary Fig. 2.
All patients
All patients
a
b
Time (months)
1.0
0.8
0.6
0.4
0.2
0.0
0
20
40
60
80
100
120
140
160
180
1.0
0.8
long course
(n=69)
long course
(n=69)
Probability of disease-free survival
0.6
Probability of overall survival
0.4
short course
(n=24)
short course
(n=24)
0.2
p=0.18
p=0.62
0.0
0
20
40
60
80
100
120
140
160
180
Time (months)

## Slide 2
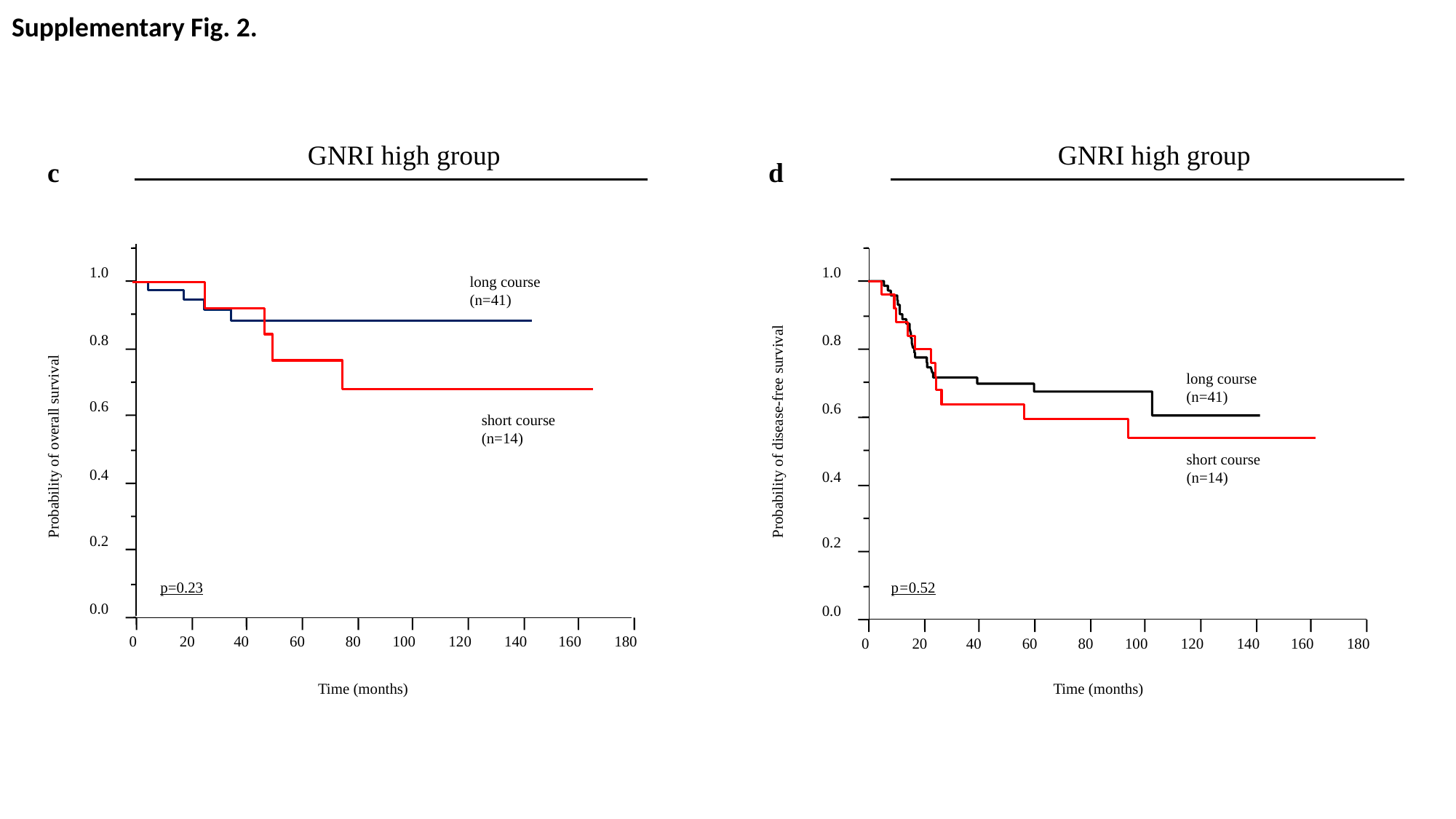

Supplementary Fig. 2.
GNRI high group
GNRI high group
c
d
1.0
0.8
0.6
0.4
0.2
0.0
0
20
40
60
80
100
120
140
160
180
Time (months)
Time (months)
1.0
0.8
0.6
0.4
0.2
0.0
0
20
40
60
80
100
120
140
160
180
long course
(n=41)
long course
(n=41)
Probability of disease-free survival
short course
(n=14)
Probability of overall survival
short course
(n=14)
p=0.23
p=0.52

## Slide 3
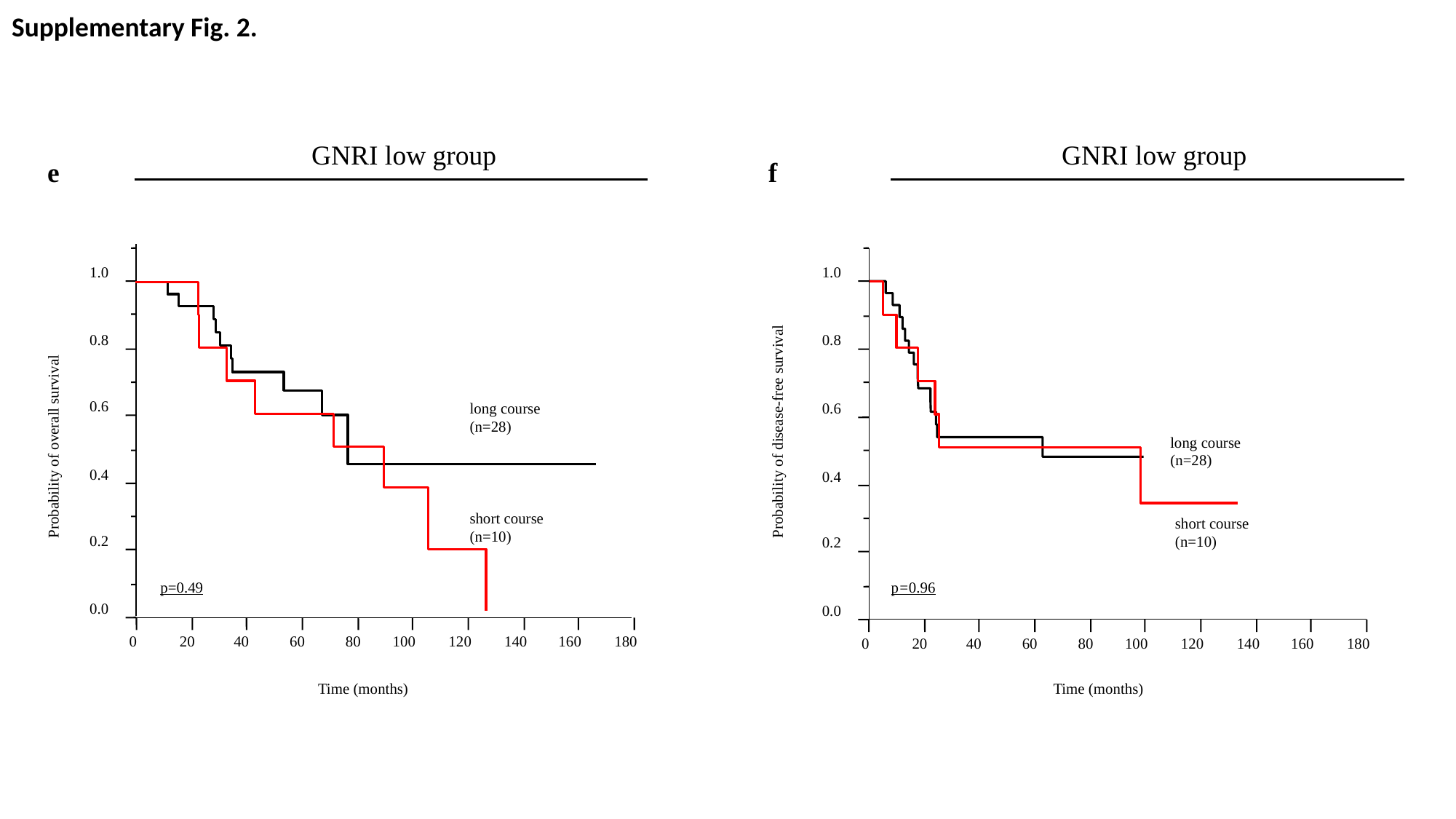

Supplementary Fig. 2.
GNRI low group
GNRI low group
e
f
1.0
0.8
0.6
0.4
0.2
0.0
0
20
40
60
80
100
120
140
160
180
Time (months)
Time (months)
1.0
0.8
0.6
0.4
0.2
0.0
0
20
40
60
80
100
120
140
160
180
Probability of disease-free survival
long course
(n=28)
Probability of overall survival
long course
(n=28)
short course
(n=10)
short course
(n=10)
p=0.49
p=0.96
