# Supplementary material for: Geriatric nutritional risk index predicts cancer prognosis in patients with local advanced rectal cancer undergoing chemoradiotherapy followed by curative surgery
Source: World J Surg Oncol. 2021 Jan 30;19:34. doi: 10.1186/s12957-021-02139-z (PMC7847581; doi:10.1186/s12957-021-02139-z)
Supplement: Supplementary file 3 — Additional file 3: Supplementary Fig. 3. Prognostic impact of serum albumin and body mass index (BMI) in patients with rectal cancer. (a) Kaplan–Meier curve for overall survival (OS) in patients with rectal cancer according to the albumin level (n = 93). OS was not significantly different between high level albumin group (n = 50) and low level group (n = 43) (p = 0.29, log-rank test). (b) Kaplan–Meier curve for disease-free survival (DFS) in patients with rectal cancer according to the albumin level (n = 93). DFS was not significantly different between high level albumin group (n = 50) and low level group (n = 43) (p = 0.25, log-rank test). (c) Kaplan–Meier curve for overall survival (OS) in patients with rectal cancer according to the BMI (n = 93). OS was significantly higher in patients with a high BMI (n = 41) compared with those with a low BMI (n = 52) (p = 0.021, log-rank test). (d) Kaplan–Meier curve for disease-free survival (DFS) in patients with rectal cancer according to the BMI (n = 93). DFS was not significantly different between high BMI (n = 50) and low BMI (n = 43) (p = 0.10, log-rank test). [file 12957_2021_2139_MOESM3_ESM.pptx]

## Slide 1
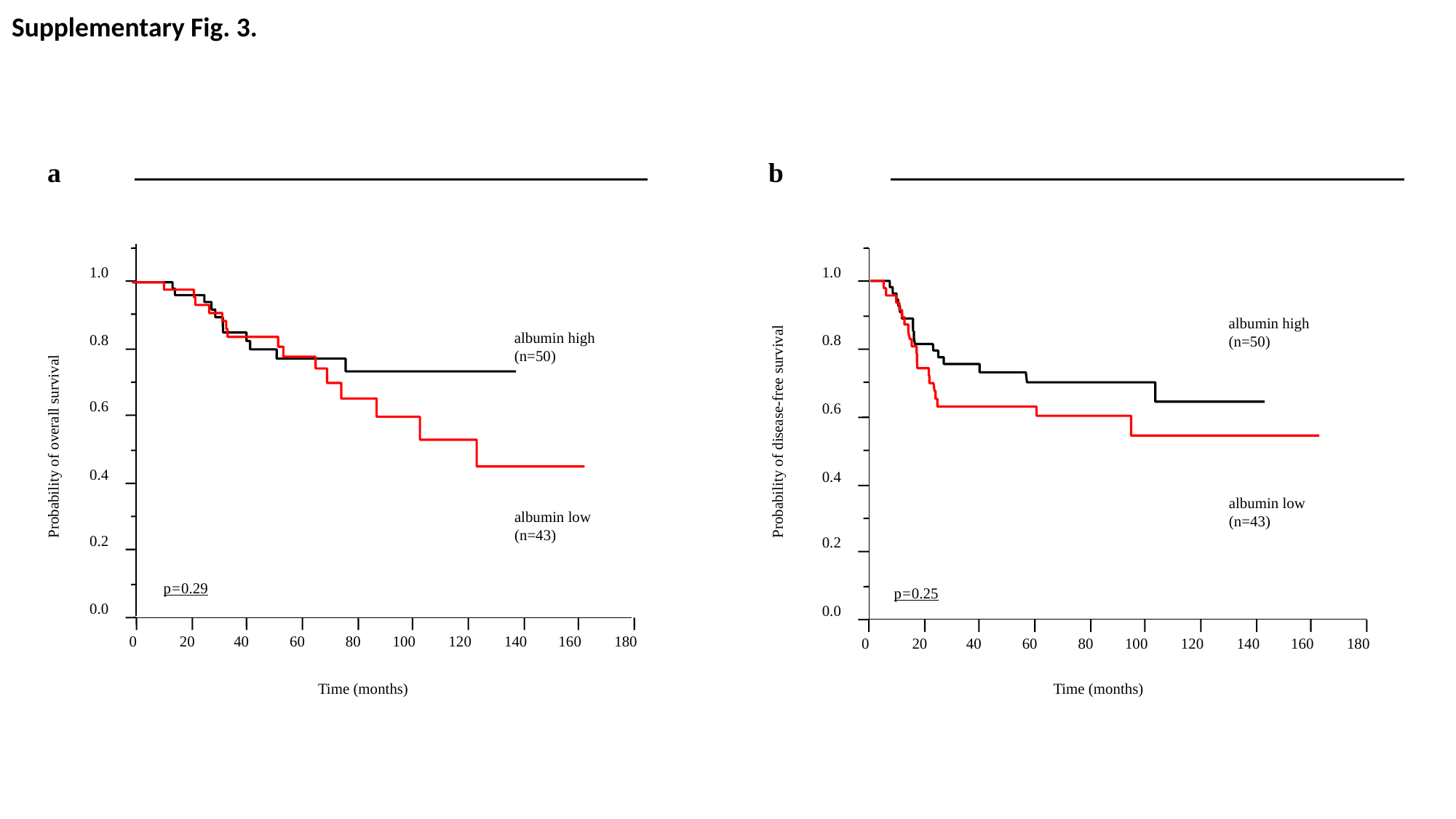

Supplementary Fig. 3.
a
b
Time (months)
1.0
0.8
0.6
0.4
0.2
0.0
0
20
40
60
80
100
120
140
160
180
1.0
albumin high
(n=50)
albumin high
(n=50)
0.8
Probability of disease-free survival
0.6
Probability of overall survival
0.4
albumin low
(n=43)
albumin low
(n=43)
0.2
p=0.29
p=0.25
0.0
0
20
40
60
80
100
120
140
160
180
Time (months)

## Slide 2
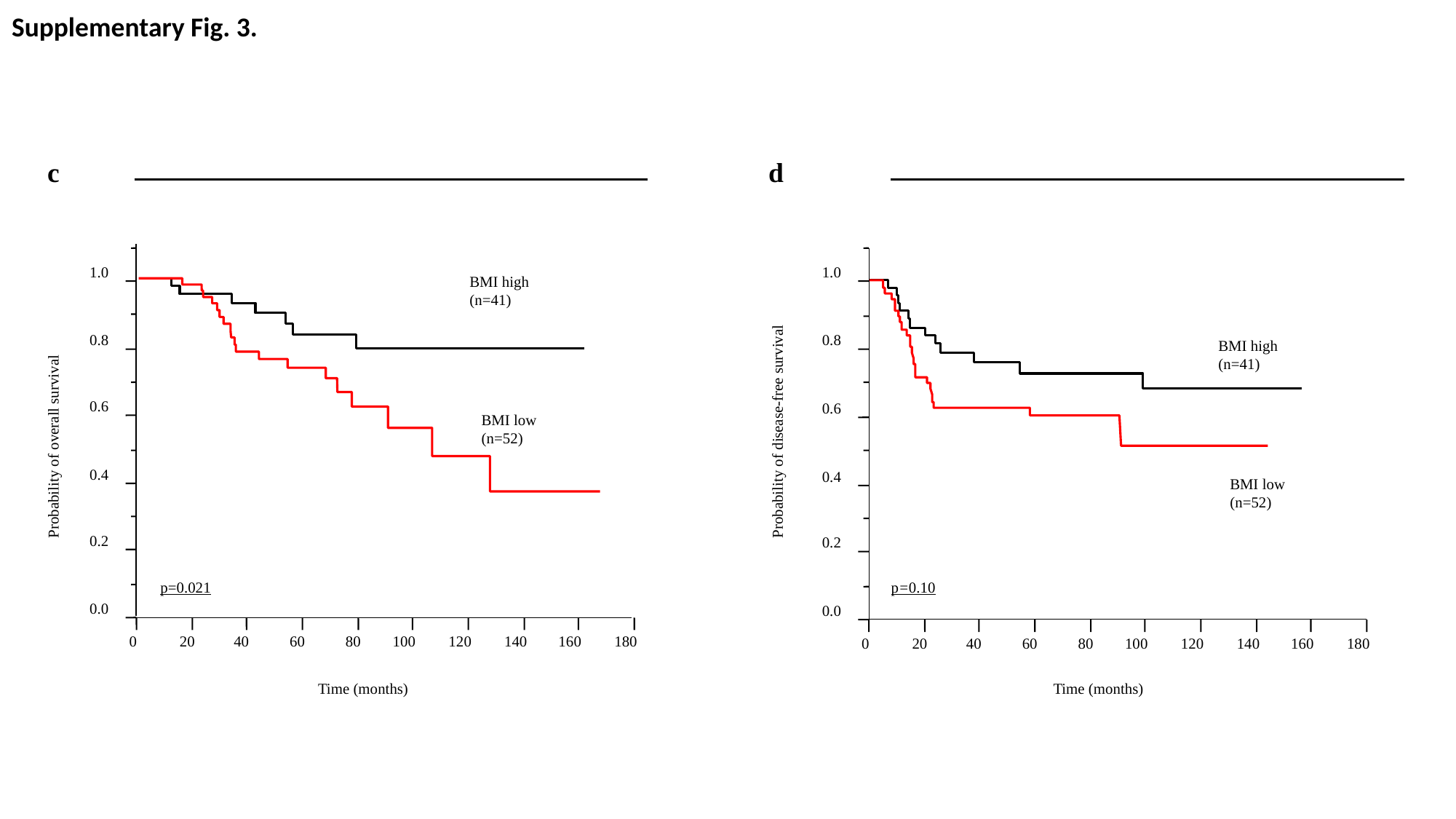

Supplementary Fig. 3.
c
d
1.0
0.8
0.6
0.4
0.2
0.0
0
20
40
60
80
100
120
140
160
180
Time (months)
Time (months)
1.0
0.8
0.6
0.4
0.2
0.0
0
20
40
60
80
100
120
140
160
180
BMI high
(n=41)
BMI high
(n=41)
Probability of disease-free survival
BMI low
(n=52)
Probability of overall survival
BMI low
(n=52)
p=0.021
p=0.10
